# Molecular survey of herpesviruses in bats from Chile and Spain reveals potentially novel species

**DOI:** 10.1007/s11259-025-10747-3

**Published:** 2025-05-01

**Authors:** Carlos Sacristán, Fernando Esperón, Irene Sacristán, Jordi Serra Cobo, Marc López Roig, Fulgencio Lisón, Javier Millán

**Affiliations:** 1 Centro de Investigación en Sanidad Animal (CISA-INIA), CSIC, Carretera Algete-El Casar de Talamanca, Km. 8,1, Valdeolmos, Madrid, 28130 Spain; 2 Veterinary Department, School of Biomedical and Health Sciences, Universidad Europea de Madrid, C. Tajo, S/N, Villaviciosa de Odón, Madrid, 28670 Spain; 3 Departament de Biologia Evolutiva, Ecologia i Ciències Ambientals, Facultat de Biologia, Universitat de Barcelona, Av. Diagonal 643, Barcelona, 08028 Spain; 4 Institut de Recerca de Biodiversitat (IRBio), Facultat de Biologia, Universitat de Barcelona, Av. Diagonal 643, Barcelona, 08028 Spain; 5 Wildlife Ecology and Conservation Lab, Zoology Department, Universidad de Concepción, Edmundo Larenas 219, Concepción, Bío Bío 4070409 Chile; 6 Instituto Agroalimentario de Aragón-IA2 (Universidad de Zaragoza-CITA), Miguel Servet 177, Zaragoza, 50013 Spain; 7 Fundación ARAID, Avda. Ranillas 1, Zaragoza, 50018 Spain; 8 One Health Institute, Facultad de Ciencias de la Vida, Universidad Andres Bello, República 440, Santiago, 8370251 Chile

**Keywords:** Emerging viruses, Europe, South America, Miniopteridae, Molossidae, Vespertilionidae

## Abstract

Bats (order Chiroptera) are known as important hosts and reservoirs for several zoonotic viruses. To this date, most virology studies in bats have focused on RNA viruses; consequently, information about DNA viruses is more limited. Herein we surveyed the presence of herpesviruses in blood or spleen samples of three bat species of Spain (n = 31) and five bat species of Chile (n = 50) by using a broad-spectrum nested PCR. Overall, herpesvirus DNA was detected in 9.7% (3/31) bats of Spain and 10.0% (5/50) bats of Chile. Three gammaherpesvirus sequence types were found in bats from Spain, while sequence types of two betaherpesviruses, two gammaherpesviruses and one unclassified herpesvirus were detected in Chilean bats, two of which could represent novel herpesvirus species. The impact (if any) of these herpesviruses on the health of the studied species needs to be clarified. This study increases our knowledge of herpesvirus diversity in bats and expands their geographic range in South America. Future herpesvirus surveillance studies are warranted to test chiropteran families other than Vespertilionidae and Molossidae in Chile and Spain.

## Introduction

The order Chiroptera (bats) is the second most diverse group of mammals, after Rodentia, and with more than 1480 species represents almost one quarter of all mammal species (Festa et al. 2023; Simmons and Cirranello 2025). Bats are particularly well-suited hosts for viral pathogens, several of which are of public health concern. These include RNA viruses of the families *Coronaviridae* (e.g., Severe acute respiratory syndrome-related coronavirus 1, Middle East respiratory syndrome-related coronavirus, and other Merbecoviruses and Sarbecoviruses), *Paramyxoviridae* (e.g., Hendra virus, Nipah virus), *Filoviridae* (Marburg virus, Ebola virus) and *Rhabdoviridae* (e.g., *Lyssavirus rabies*) (Hayman 2016; Cui et al. 2019; Letko et al. 2020), among others. Most virology studies in bats have focused on RNA viruses; however, information about DNA viruses is more restricted.

One of the most important DNA viral families is *Orthoherpesviridae* (order *Herpesvirales*), which comprises enveloped double-stranded DNA viruses able to establish latency in their natural hosts, subdivided into three subfamilies: *Alphaherpesvirinae*, *Betaherpesvirinae* and *Gammaherpesvirinae* (Davison 2010). These viruses usually co-diverge with their natural host species, giving rise to an elevated number of species (Kaján et al. 2020). Due to the large number of bat species, a high quantity of herpesvirus species is also expected. Additionally, their colonial behavior could favor viral transmission (Serra-Cobo and López-Roig 2017). To date, 348 alpha-, beta-, and gammaherpesviruses and unclassified herpesviruses have been discovered in 75 bat species worldwide, according to the zoonotic and vector-borne viruses database (Zhou et al. 2022). Some species have been recognized by the International Committee on Taxonomy of Viruses, classified into the subfamilies *Alphaherpesvirinae* (genus *Simplexvirus*), *Betaherpesvirinae* (genus *Quwivirus*) and *Gammaherpesvirinae* (genera *Percavirus* and *Patagivirus*) (Gatherer et al. 2021). Nevertheless, herpesvirus surveillance studies are not uniform, and the information available from certain geographical areas is scarce. In Spain, there is only one study about herpesviruses in bats (Pozo et al. 2016), while no data are available from Chile. Indeed, reports of herpesvirus in bats from South America are still scarce (Salmier et al. 2017; Moreira Marrero et al. 2021). Our goal was to survey and identify herpesviruses in different species of bats captured in Spain and Chile.

## Materials and methods

### Field methods

We used a convenience sampling approach. In Spain, 31 bats belonging to two different families were studied. Most of the sampled bats belonged to the family Miniopteridae—Schreibers’s long-fingered bats (syn. Schreiber's bent-winged bat *Miniopterus schreibersii*, n = 28), whereas the other three belonged to the family Vespertilionidae, i.e., greater mouse-eared bats (*Myotis myotis*, n = 2) and long-fingered bat (*Myotis capaccinii*, n = 1, Table 1). The animals were captured using nets in an abandoned mine (41°17’N, 1°47’E) and two caves (41°38’N, 2°44’E and 42°1’N, 0°57’E) of Catalonia, Spain, in the boreal summer (July 2-August 26) of 2013.

In Chile, we captured 50 bats of four species within the families Molossidae -Brazilian free-tailed bat (*Tadarida brasiliensis*, n = 14), and Vespertilionidae—Chilean myotis (*Myotis chiloensis*, n = 17), cinnamon red bat (*Lasiurus varius*, n = 7), hairy hoary bat (*Lasiurus villosissimus*, n = 1), and small big-eared brown bat (*Histiotus montanus*, n = 11, Table 1). The Brazilian free-tailed bats were captured in the Metropolitan Region of Santiago (33°12′00’’S, 70°41′00’’W) in 2014 for a rabies surveillance program. The remaining Chilean bat species were captured in the forest of Antuco (Biobío province, 37º23′39’’S, 71º24′32’’W) on November 10–11, 2017 and in the Parque Ecológico y Cultural of Rucamanque, Temuco (Araucanía province, 38º40′06’’S, 72º36′17’’W), on December 31, 2018, in Chile, and released after sampling (Table 1). The studied animals belong to the families Vespertilionidae and Molossidae, which along with Emballonuridae, are the only three bat families shared between the Old and New Worlds (Peixoto et al. 2018). The sex and age class of the bats sampled in Spain and Chile are displayed in Table 1.

**Table 1:** Biological data of the adult bats sampled in Spain and Chile

Common name	Scientific name	n	Age class	Sex		Location	Country
				M^a^	F^b^		
Schreibers’s long-fingered bat	*Miniopterus schreibersii*	28	Adult	18	10	Avenc l'Esquerrà cave (Barcelona, n = 9), Montsec cave (Lérida, n = 8), Malgrat mine (Barcelona, n = 11)	Spain
Greater mouse-eared bat	*Myotis myotis*	2	Adult	-	2	Malgrat mine	Spain
Long-fingered bat	*Myotis capaccinii*	1	Adult	-	1	Avenc l'Esquerrà cave	Spain
Chilean myotis	*Myotis chiloensis*	17	Adult	2	15	Antuco (Biobío, n = 16) and Rucamanque-Temuco (La Araucanía, n = 1)	Chile
Cinnamon red bat	*Lasiurus varius*	7	Adult	3	4	Rucamanque-Temuco	Chile
Hairy hoary bat	*Lasiurus villosissimus*	1	Adult	1	-	Rucamanque-Temuco	Chile
Small big-eared brown bat	*Histiotus montanus*	11	Adult	2	9	Rucamanque-Temuco	Chile
Brazilian free-tailed bat	*Tadarida brasiliensis*	14	N.A.^a^	N.A	N.A	Metropolitan Region of Santiago	Chile

*M* male; *F* female; ^a^N.A = not analyzed

All animals were handled according to good animal welfare practices, as defined by European and Chilean legislation. For all Vespertilionidae and Miniopteridae bats, blood samples (50 µl) were collected from the cephalic vein, placed in ethylenediaminetetra-acetic acid (EDTA) tubes, and maintained at − 20 °C until analysis. These bats were released after sampling at the capture site. The Molossidae bats– Brazilian free-tailed bats– were euthanized, necropsied and sampled as part of the national rabies surveillance program; samples were kept frozen at − 20 °C until analysis. Bat captures and sampling in Spain and Chile were performed with the permission of the Spanish Regional Committee for Scientific Capture (Permit number 2013: SF/555) and the Servicio Agrícola y Ganadero (SAG-Chile. Resolución exenta Nº8431/2021) of the Chilean Government, respectively.

### Molecular methods

Total DNA was extracted from frozen blood of 31 bats from Spain using the QuickGene Mini 80 nucleic acid isolation machine (QuickGene, Kurabo, Japan), and from 36 blood samples and 14 spleen samples of bats of Chile with the DNeasy Blood & Tissue kit (Qiagen, Hilden, Germany), according to the manufacturers’ instructions. Subsequently, DNA was tested for herpesvirus using the broad-spectrum PCR protocol described by VanDevanter et al. (1996), able to amplify a 230–330 bp fragment of the catalytic subunit of the DNA polymerase gene of alpha-, beta- and gammaherpesviruses. All amplicons of the expected size were purified with ExoSAP-IT (Affymetrix Inc., Santa Clara, USA) and directly sequenced in both directions. The obtained sequences were assembled in MEGA 7 by ClustalW alignment, to construct the consensus sequences. After that, a BLASTn search was performed to compare the obtained consensus sequences to those available in GenBank/DDBJ/EMBL database. The genetic nucleotide (nt) and amino acid (aa) distances between the obtained consensus sequences and the most similar retrieved from the GenBank/DDBJ/EMBL database were calculated in MEGA 7 based on p-distance.

For phylogenetic analysis, a nucleotide maximum likelihood phylogenetic tree was constructed using MEGA 11.0 with a bootstrap value of 1000 with a General Time Reversible model with inversions and Gamma distribution including the sequences obtained in this study, and representative mammal alpha-, beta- and gammaherpesvirus sequences of viral species of genera recognized by the International Committee on Taxonomy of Viruses. Bootstrap frequencies lower than 70 were omitted. The obtained herpesvirus species were submitted to GenBank under accession numbers PQ014587-PQ014594.

## Results

### Spain

Three out of 31 (9.7%) bats from Spain were herpesvirus-positive, all of which were Schreibers’s long-fingered bats captured in the same abandoned mine of Catalonia (n = 2, cases SP-535 and SP-541) or in the cave of Avenc l'Esquerra (n = 1, case SP-534). The obtained sequence types of cases SP-535 and SP-541 were similar between them (nt and aa similarities of 98.4% and 96.9%, respectively). When compared to SP- 534, they had identities over 96% (similarities of 97.9% nt and 100% aa between SP-535 and SP-534, and of 96.4% nt and 96.9% aa between SP-541 and SP-534). The retrieved nucleotide sequences were more similar (96.4% to 97.6%) to a gammaherpesvirus sequence (KR261846) described in Schreibers’s long-fingered bat in China, while the deduced amino acid sequences were closer (98.2%) to gammaherpesvirus sequences found in that Schreibers’s long-fingered bat (KR261846) and in a least horseshoe bat (*Rhinolophus pusillus blythi*, KR261852), also from China (Table 2).

**Table 2:** Nucleotide and amino acid identities of the herpesvirus sequences detected in bats from Spain and Chile with the closest ones from the GenBank/EMBL/DDBJ database

ID	Host species and year of sampling	GenBank accession nº	Nucleotide similarity and query cover	Amino acid similarity and query cover
SP- 534	*Miniopterus schreibersii* 2013	PQ014587	97.6% to γ-HV of a Schreibers's long-fingered bat (*Miniopterus schreibersi*) (KR261846) sampled in 2011 and of a least horseshoe bat (*Rhinolophus pusillus blythi*, KR261852) sampled in 2013, both of China. 100% query cover	98.2% to γ-HV sequences of a Schreibers's long-fingered bat (KR261846) and of a least horseshoe bat (KR261852), both of China. 100% query cover
SP- 535	*Miniopterus schreibersii* 2013	PQ014588	96.4% to γ-HV of a Schreibers's long-fingered bat (KR261846) and of a least horseshoe bat (KR261852), both of China. 100% query cover	
SP- 541	*Miniopterus schreibersii* 2013	PQ014589	95.2% to γ-HV of a Schreibers's long-fingered bat of China (KR261846) and of a least horseshoe bat (KR261852), both of China. 100% query cover	96.4% to γ-HV sequences of a Schreibers's long-fingered bat (KR261846) and of a least horseshoe bat (KR261852), both of China. 100% query cover
ID983	*Tadarida brasiliensis* 2014	PQ014590	100% to γ-HV of a Brazilian free-tailed bat (*Tadarida brasiliensis*) from Uruguay (MT876198) sampled in 2015. Our sequence is slightly longer than the one from Uruguay (192 vs. 170 bp). 88% query cover	100% to γ-HV of a Brazilian free-tailed bat (*Tadarida brasiliensis*) from Uruguay (MT876198). 89% query cover
ID985	*Tadarida brasiliensis* 2014	PQ014591	97.7% to a β-HV sequence from a bat of Uruguay (LC578846) sampled in 2015, identified as Argentinian brown bat (*Eptesicus furinalis*) in GenBank and as *Myotis* sp. in the manuscript Moreira et al. (2021) Our sequence is slightly longer than the one of Uruguay (195 vs. 173 bp). 88% query cover	94.7% to the same β-HV sequence described on the nt comparison. 89% query cover
ID986	*Tadarida brasiliensis* 2014	PQ014592	Highly divergent, best similarity (59.8%) with alphaherpesvirus (Pacific white-sided dolphin (*Lagenorhynchus obliquidens*)) of Japan (AB747558), likely sampled in 2013. 44% query cover	69.1% to two betaherpesvirus sequences of intermediate leaf-nosed bat (*Hipposideros larvatus*) of China (OR998958 and OR998959), with 100% query cover. 48.4% to alphaherpesvirus (Pacific white-sided dolphin (*Lagenorhynchus obliquidens*) of Japan (AB747558) and with an alphaherpesvirus of a striped dolphin (*Stenella coeruleoalba*) of Spain (KP995681), the latter sampled in 2011. 98% query cover
ID987	*Tadarida brasiliensis* 2014	PQ014593	94.4% to a β-HV sequences of Brazilian free-tailed bat of Uruguay (MT906861) sampled in 2015. 99% query cover	95.3% to the same β-HV sequence described on the nt comparison. 100% query cover
ID992	*Tadarida brasiliensis* 2014	PQ014594	82.4% to a γ-HV of a Brazilian free-tailed bat of Uruguay (MT906862) sampled in 2015. Our sequence is slightly longer than the one of Uruguay 194 vs 187). 95% query cover	88.7% to a γ-HV of a Brazilian free-tailed bat of Uruguay (MT906862). 100% query cover

### Chile

Overall, five out of 50 (10.0%) bats from Chile were positive (CL-983, CL-985, CL-986, CL-987, and CL-992), all of them Brazilian free-tailed bats. We identified two betaherpesviruses (CL-985, CL-987), and two gammaherpesvirus (CL-983, CL-992) sequence types; while it was not possible to assign the herpesvirus identified in one of the animals (CL-986) to any herpesviral subfamily. The most similar sequences for all of them were found in Brazilian free-tailed bats sampled in Uruguay, with the exception of the highly divergent herpesvirus found in CL-986, which presented the highest nucleotide identity (58.9%) with sequences found in cetaceans, and the highest amino acid identity (69.1%) with two betaherpesvirus sequences of intermediate leaf-nosed bat (*Hipposideros larvatus*) of China (OR998958 and OR998959) (Table 2).

**Fig. 1:**
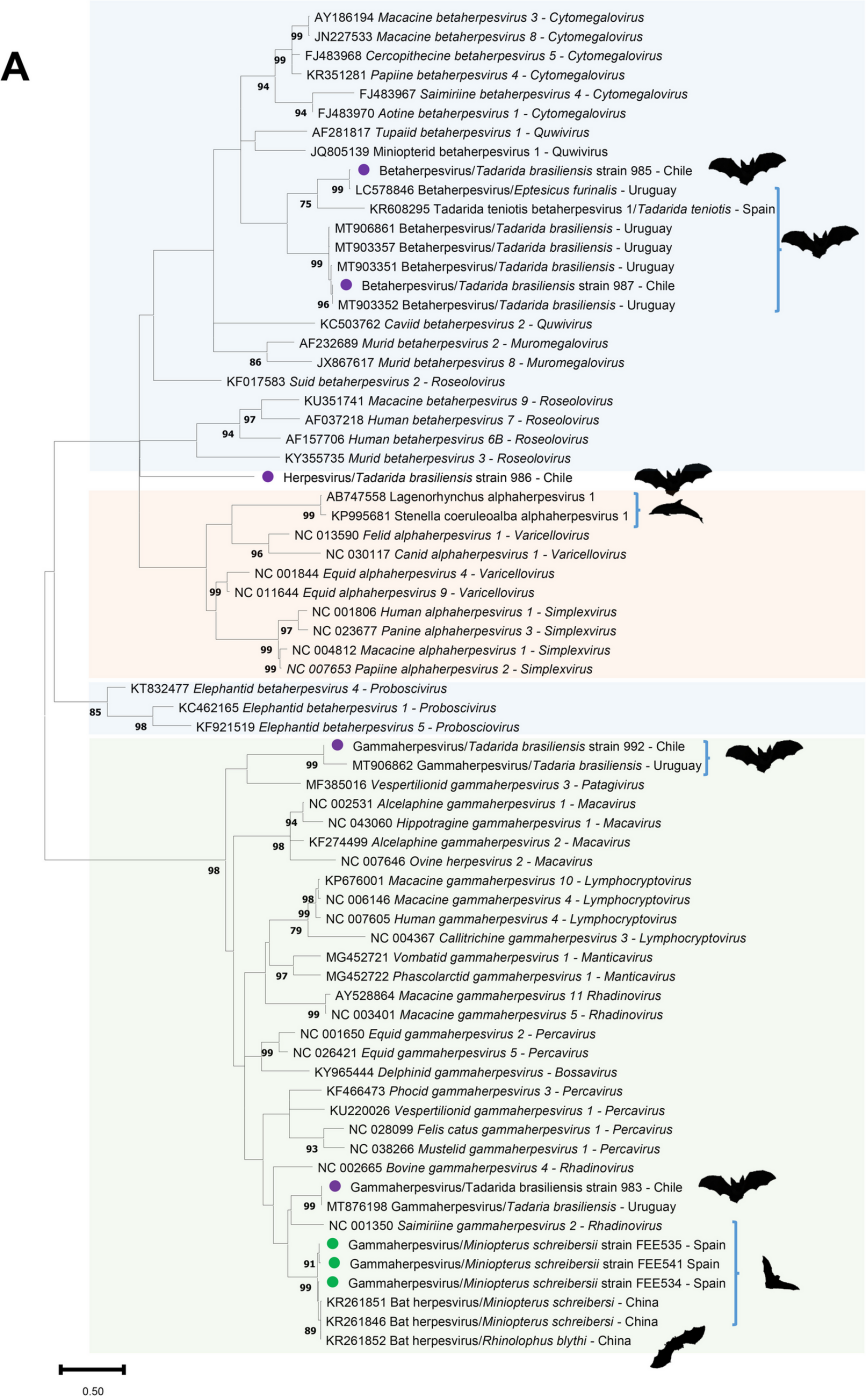

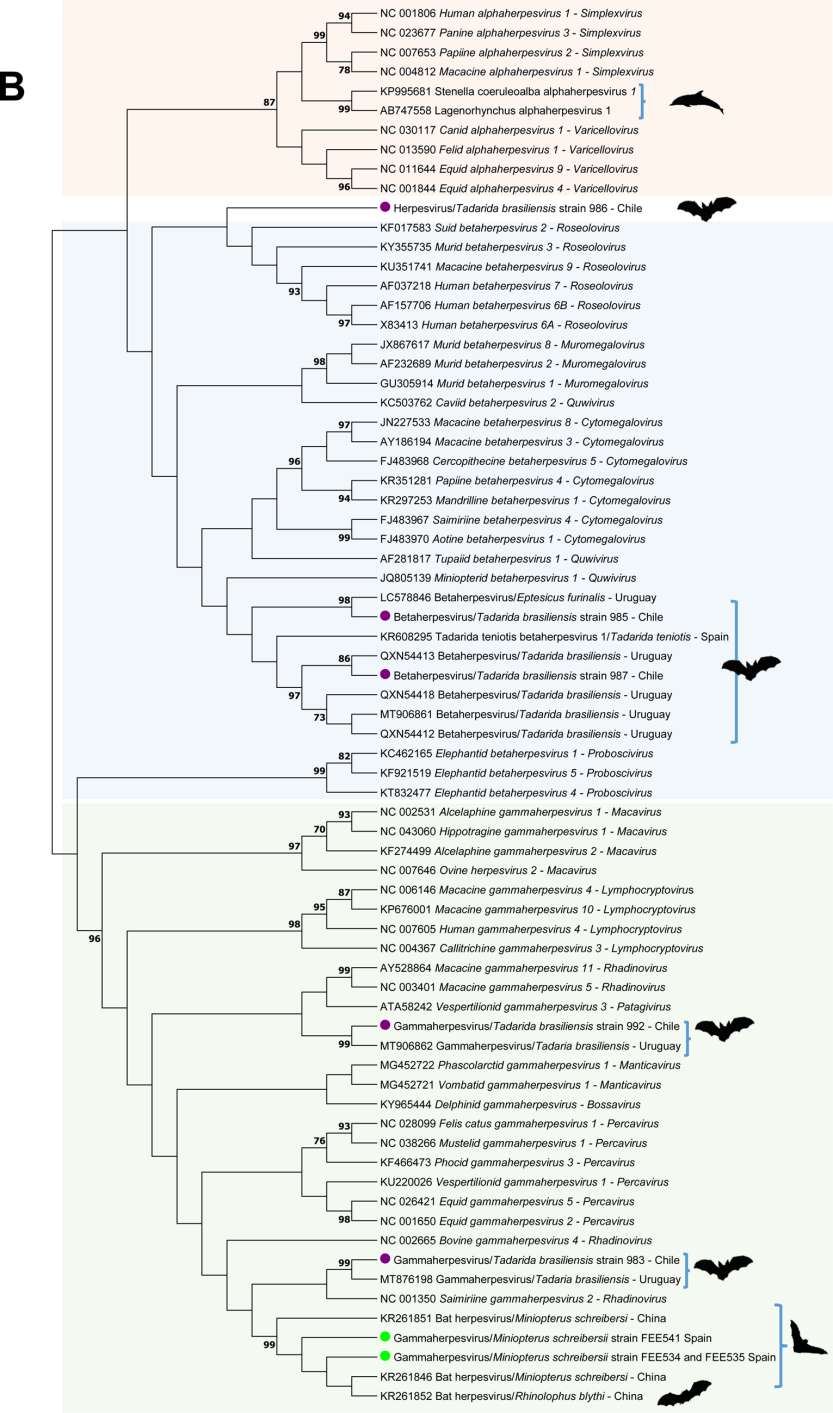
Maximum likelihood phylogenetic trees based on the (**A**) General Time Reversible model with inversions, gamma distribution and 1000 replicates of the nucleotide herpesvirus DNA polymerase sequences (1) retrieved from bats of Spain (green dots and silhouettes) and Chile (purple dots and silhouettes), (2) similar bat and dolphin herpesvirus sequences (marked with silhouettes), and (3) representative herpesviral species of all the herpesviral genera with the three *Orthoherpesviridae* subfamilies (*Alphaherpesvirinae*, *Betaherpesvirinae* and *Gammaherpesvirinae*) recognized in mammals by the International Committee on Taxonomy of Viruses; and (**B**) Le Gascuel model, discrete gamma distribution and 1000 replicates of the deduced amino acid herpesvirus DNA polymerase sequences identified in the same species selected for tree in Fig. 1.A. The trees with the highest log likelihood are shown. Bootstrap values < 70 were omitted. The country of origin of the sequences identified in bats was specified. The *Alphaherpesvirinae*, *Betaherpesvirinae* and *Gammaherpesvirinae* subfamilies are marked with orange, blue and green boxes, respectively

The phylogram accurately reconstructed the phylogeny of most of the selected herpesvirus genera. According to our results, none of the herpesviral sequences obtained in this study were classified into the herpesviral genera previously described in mammals (Fig. 1).

## Discussion

We detected three different gammaherpesvirus sequence types in Schreibers’s long-fingered bats of Spain. Our gammaherpesvirus nucleotide sequences were more similar to a sequence described in bats in China (Zheng et al. 2016). These samples were allegedly obtained in Schreibers’s long-fingered bat, although they can belong to different bat species, as according to the IUCN Schreibers’s long-fingered bats are present in multiple European countries, Turkey, the Near East, and North Africa, but not in the Far East (Cistrone et al. 2023). Regarding the deduced aa sequences, they were more similar to gammaherpesviruses from the aforementioned Schreibers’s long-fingered bats and a least horseshoe bat (*Rhinolophus pusillus blythi*) of China (Zheng et al. 2016). Gammaherpesviruses are generally considered host-specific (Azab et al. 2018); the Schreibers’s long-fingered bat could be the natural host of the detected sequences. Additionally, it should be noted that Schreibers’s long-fingered bat is a migratory species, able to perform long-distance movements, which could contribute to the spread of herpesviruses within different populations. According to DBatVir (2024), Spain is the European country with the highest number of viral descriptions in bats; however, only 4 out of the 298 viruses reported in this country were herpesviruses, which could indicate a lack of studies (DBatVir 2024). Nevertheless, a high number of herpesvirus-positive bats (168) was reported by Pozo et al. (2016) in the only herpesvirus survey performed to this date in Spain. The prevalence observed in Schreibers’s long-fingered bat in our study (10%, 3/30) is lower than the one (72.5%, 29/40) reported in the same species by Pozo et al. (2016). These differences could be attributed to dissimilitude in the techniques selected for herpesvirus detection.

Herein we tested 5 of the 13 bat species present in Chile (Rodríguez-San Pedro et al. 2016), and identified two betaherpesvirus, two gammaherpesviruses and an unassigned orthoherpesvirus. To the authors’ knowledge, this is the first report of herpesviruses in bats of Chile. Until now, the only known viruses in Chilean bat species were rhabdoviruses (DBatVir 2024). Most of the obtained sequence types retrieved in the Brazilian free-tailed bat of Chile were more similar to those obtained in bats of the same species of Uruguay (Moreira Marrero et al. 2021); the exception was a highly divergent herpesvirus, closest– but with low similarity– to alphaherpesvirus sequences described in cetaceans. This herpesvirus is likely a novel species. One of the gammaherpesviruses detected in a Brazilian free-tailed bat could also be a novel species, as it presented nucleotide and amino acid identities of only 82.4 and 88.7%, respectively, compared with the closest sequence, identified in Brazilian free-tailed bat of Uruguay. The herpesvirus occurrence (89.3%, 25/28) detected in Brazilian free-tailed bats from Uruguay by Moreira Marrero et al. (2021) was higher than the one observed in our study (10.4%, 5/48). This study expands the herpesvirus geographic range in bats of South America.

In summary, we detected novel beta- and gammaherpesvirus sequence types and a divergent unassigned herpesvirus in bats of Spain and Chile, some of which are likely novel species. This study expands the geographic range of herpesviruses in bats of South America. The impact (if any) of these herpesviruses on the health of the studied species needs to be clarified. Future herpesvirus surveillance studies are warranted to test chiropteran families other than Vespertilionidae and Molossidae in Chile and Spain.

## Data Availability

All data are available in the manuscript. The datasets generated and/or analyzed during the current study are available in the GenBank repository (accession numbers PQ014587-PQ014594).

## Declarations

### Permits

Bat captures and sampling in Spain and Chile were performed with the permission of the Spanish Regional Committee for Scientific Capture (Permit number 2013: SF/555) and the Servicio Agrícola y Ganadero (SAG-Chile. Resolución exenta Nº8431/2021) of the Chilean Government, respectively.
